# The Impact of a Challenge-Based Learning Experience in Physical Education on Students’ Motivation and Engagement

**DOI:** 10.3390/ejihpe13040052

**Published:** 2023-03-26

**Authors:** Luis Simón-Chico, Alba González-Peño, Ernesto Hernández-Cuadrado, Evelia Franco

**Affiliations:** 1 Facultad de Ciencias Humanas y Sociales, Universidad Pontificia Comillas, C/Universidad Comillas, 3-5, 28108 Madrid, Spain; 2 Facultad de Ciencias de la Actividad Física y del Deporte—INEF, Universidad Politécnica de Madrid, C/Martín Fierro, 7, 28040 Madrid, Spain; 3 I.E.S. María Pacheco, Av. de Barber, 4, 45004 Toledo, Spain

**Keywords:** challenge-based learning, badminton, self-determination theory, innovative methodology, circumplex approach

## Abstract

The present study investigated how challenge-based learning (CBL) in physical education (PE) may affect students’ basic psychological needs (BPNs), motivational regulations, engagement, and learning in comparison with a traditional teaching (TT) methodology. A quasiexperimental study with experimental and control groups was carried out. In total, 50 participants (16 boys and 34 girls) between 13 and 15 years old (M_age_ = 13.35, SD = 0.62) were involved in the experience for 6 weeks (n_control_ = 24; n_experimental_ = 26). Validated questionnaires were administered both before and after the intervention in both groups. Furthermore, theoretical knowledge and badminton-specific motor skill tests were carried out in both groups after the intervention. An analysis showed that after the intervention, students in the CBL condition improved their autonomy (M_before_ = 3.15 vs. M_after_ = 3.39; ES = 0.26 *), competence (M_before_ = 4.01 vs. M_after_ = 4.18; ES = 0.33 *), and relatedness satisfaction (M_before_ = 3.86 vs. M_after_ = 4.06; ES = 0.32 *). As for behavioural engagement measures, students in the CBL condition exhibited higher scores after than those from before (M_before_ = 4.12 vs. M_after_ = 4.36; ES = 0.35 *). No significant changes were observed for motivational regulations or agentic engagement. On learning outcomes, students in the experimental group achieved higher scores in both theoretical knowledge (M_contol_ = 6.48 vs. M_experimental_ = 6.79) and badminton-specific motor skills (M_contol_ = 6.85 vs. M_experimental_ = 7.65) than the control group did. The present study findings highlight that CBL might be a valid and effective methodological approach for students in PE to achieve adaptive motivational, behavioural, and learning outcomes.

## 1. Introduction

Student learning in physical education (PE) is a concern shared by the educational community, and motivation and commitment are two of the factors that seem to be key determinants in the teaching–learning process. According to Ryan and Deci [1], the more adaptive the motivation experienced by students is, the more willing students are to engage in certain behaviours and the more they learn. Through the lens of self-determination theory (SDT) [2], an important research line has focused on the mechanisms explaining how interactions affect several behaviours in general health contexts [3] and, in particular, teacher–student interactions in the PE context [4]. According to these authors, motivation can be described as the process that moves a person to act in a certain way. Within this theory, several types of motivations are differentiated as part of a continuum according to the degree of self-determination, which are identified as autonomous motivation, controlled motivation, and amotivation. Autonomous motivation is manifested by experiencing intrinsic motivation, which refers to the inherent reason to do something for pleasure, and identified regulation, which is characterized by engagement with a behaviour that seems to be beneficial to the person. Between intrinsic motivation and amotivation, controlled motivation is manifested by experiencing introjected motivation, which may occur when the person feels guilty for not performing a certain action, and external motivation reflects when a person is moved only by an external reward offered to them. Finally, amotivation happens when the person cannot find a reason to engage in a certain behaviour. SDT also proposes that people have three innate basic psychological needs (BPNs), namely autonomy, competence, and relatedness, which are associated with this self-determined form of motivation. In PE, autonomy is associated with the feeling of freedom to make decisions during the learning process and to express one’s own sentiments and opinions. Competence refers to the perception of feeling capable of facing the proposed activities and, thus, to a feeling of achievement. Relatedness refers to feelings of belonging to the group [5,6]. There is strong evidence that the satisfaction of these needs in a PE context will lead to desirable outcomes, such as autonomous motivation and both behavioural and emotional engagement [7]. Although basic psychological needs satisfaction has been more thoroughly studied, highlighting the importance of their fulfilling to enhance adaptive behaviours, Ryan and Deci [8] proposed that the thwarting of basic psychological needs will lead to nonoptimal development and ill-health. Basic psychological needs frustration is thought to occur when individuals perceive their psychological needs to be actively undermined in their close social environment [6].

### 1.1. Teachers’ Needs-Supportive and Needs-Thwarting Behaviours in Physical Education

In the PE context, first, teachers can support autonomy by showing interest in students’ feelings and preferences, offering them a relevant space for decision-making, or fostering a climate in which they can freely express their sentiments [9]. Second, teachers can support their students’ competence in the classroom by providing structure both before the activity, such as by setting clear expectations or adapting tasks to the students’ level of skill, and during the activity, such as by providing effective feedback and thus guiding the learning process [10]. Lastly, relatedness support is characterized by the creation of warm contexts where teachers are empathetic, caring, and understanding of their students [11].

On the other hand, when displaying needs-thwarting behaviours, teachers exercise power as an authority by ignoring students’ perspectives or interests, demanding respect, or pressuring students by referring to their self-confidence [12]. It has been suggested that reducing these controlling behaviours will foster more adaptative outcomes between students [13,14]. In this line, a recent study has proposed a classification system to identify different teachers’ motivational behaviours consistent with SDT [15]. In this study, teachers’ behaviours have been organized by psychological needs and by how they affect them (whether supporting or thwarting needs satisfaction).

### 1.2. Students’ Engagement in Physical Education

Students’ motivation in PE seems to be closely related to their engagement in the subject. Students’ engagement is a multifaceted concept that reflects behavioural, emotional, and cognitive aspects [16]. When students are engaged in the class, they listen, strive and persist in the task, answer the questions that the teacher asks, and/or enjoy engaging in the proposed activities [17,18]. Behavioural engagement has emerged as an important construct in the prediction of students’ performance and learning achievement [17,19,20,21,22], and it can be nurtured by needs-supportive teaching [23,24,25]. Recent works have also pointed out the relevant role that agentic engagement plays in the improvement of one’s learning, development, and performance [26,27]. This type of engagement has been defined as what students say and do to create a more motivationally supportive learning environment for themselves [28,29]. Some expressions of agentic engagement in students include asking questions, expressing preferences, or asking for guidance and support [26]. It has even been found that students’ engagement can influence teachers’ motivating styles, specifically autonomy-supportive teaching [30].

### 1.3. The Association between Methodological Approaches and Students’ Motivation and Behaviour in Physical Education

Unsurprisingly, both motivational and behavioural processes among students in the PE setting are likely to be affected by interactions happening in class [31,32]. In this regard, the use of certain methods in which the focus has shifted from the teacher and instruction to the student and learning have been found to be associated with more-adaptive motivational regulations [33,34,35] and with higher engagement [36] in PE. More specifically, there are certain specific methodological approaches that have been suggested to be successful in the creation of adaptive contexts. This is the case for the sport education model (SEM) [37]; the hybridization of the SEM and teaching games for understanding (TGfU) [38]; practice and inclusion teaching styles [39]; and cooperative learning (50). These studies highlight the important role that methodological approaches play in students’ motivation, especially those with low-motivation profiles [35,40], which could in turn help to achieve greater student involvement and adherence to sports practice [41].

### 1.4. The Potential of Challenge-Based Learning to Improve Students’ Motivation and Engagement

Challenge-based learning (CBL) is a learning framework that has been described in multiple ways [42]. According to Nichols, Cator, and Torres [43], it is a learning methodology that consists of posing a challenge as a didactic element, thus promoting the learning of knowledge together with enriching, attractive, motivating, and meaningful experiences for students. In CBL, it is important to progressively give autonomy to the student, and it is necessary to propose stimulating content that is focused on both the product and the process of learning. The teacher must act as a guide, expert, stimulator, and supporter of the learner, whose characteristics must be considered when specifying and defining learning objectives. As for the learning content, it should provide students with the opportunity to interact with one another. This educational framework has been investigated in different disciplines, with an increasing emphasis in the fields of engineering and medicine but few studies in the field of PE [42,44,45].

Franco, Martínez-Majolero, Almena, and Trucharte [46], developed a proposal for the implementation of CBL within physical-activity-related educational contexts. According to this proposal, the adaptation of the complexity of the challenges to the students, the design of well-structured activities, the encouragement of cooperation among students, and the establishment of appropriate evaluation methods could be key elements of the successful implementation of CBL in the aforementioned contexts [46].

The differential elements of the CBL according to the proposal by Franco, Martínez-Majolero, Almena, and Trucharte [46] concern the methods used, the teaching strategies, and teaching techniques, as well as the teaching styles, the groupings, the implementation of individualization, specific features in task presentation, the provision of students’ autonomy support, the teacher’s role, the students’ involvement in their own evaluation, the presence of collaborative work, and the use of ICTs (see Appendix A, Table A1).

When analysing these features in detail, it can be perceived how some of those characteristics resemble features of a needs-supportive style, according to the descriptions provided by Ahmadi, Noetel, Parker, Ryan, Ntoumanis, Reeve, Beauchamp, Dicke, Yeung, Ahmadi, et al. [15] and according to previous studies (e.g., [47]) More specifically, when implementing CBL, teachers are likely to implement strategies that support autonomy, such as allowing students to choose tasks by providing a variety of activities, letting students manage their own cognitive load, and provoking curiosity to facilitate exploratory behaviours. Teachers can also support competence by setting the right amount of challenge, clarifying the path towards goal achievement, providing structure so that students clearly understand what is expected, fostering a deeper understanding, providing opportunities for accurate self-reflection of effort and progress, or allowing each student hands-on practice to progress their development of a skill. Finally, teachers can implement strategies to support relatedness by showing care and encouraging students to express themselves, allowing students to work with people with similar interests, or promoting cooperation towards a goal.

### 1.5. The Present Study

There is previous evidence of the implementation of certain methodologies that can improve students’ motivational and behavioural outcomes in the PE context. Furthermore, several attempts have been made to incorporate CBL into higher education. However, there is no evidence of the effect of this approach on secondary education students’ motivation and engagement in the PE context.

The present study aims to analyse how CBL could affect students’ BPN satisfaction and frustration, motivation, behavioural engagement, and agentic engagement in comparison with a traditional teaching (TT) methodology. This study thus adds to the existing literature by answering the following question: are there differences in students’ motivation and engagement according to the methodology that they follow in class (CBL vs. TT). Thanks to the features of CBL, it is hypothesized that a CBL-based experience can positively affect adaptive students’ motivational (BPN satisfaction and more-autonomous forms of motivation) and behavioural outcomes (behavioural and agentic engagement) and can prevent maladaptive students’ motivations (BPN frustration and amotivation and controlled forms of motivation). Lastly, this investigation aimed to check whether students’ performance in theoretical knowledge and their practical competence acquisition differ according to the methodology used in class (CBL vs. TT). In this regard, it was hypothesized that methodology might not affect theoretical knowledge acquisition. However, it was expected that the group taught through CBL would reach higher levels of practical competence given that these students would be engaged in a more individualized experience.

## 2. Materials and Methods

### 2.1. Participants

The sample comprised 50 students (16 boys and 34 girls) between the ages of 13 and 15 years (M = 13.35, SD = 0.62) from two secondary education school classes in Toledo (Spain). The classes participating in the study were randomly selected from among those at educational level that the teacher taught. All the students were in their third year of secondary education. One group with 24 students was assigned to a control group, and another group with 26 students was assigned to an experimental group.

### 2.2. Instruments

#### 2.2.1. Basic Psychological Needs Satisfaction and Frustration

Students’ perception of basic psychological needs satisfaction and frustration was assessed by using a Spanish version, adapted to the PE context [48], of the scale designed by Chen, Vansteenkiste, Beyers, Boone, Deci, Van der Kaap-Deeder, Duriez, Lens, Matos, Mouratidis, et al. [49]. The stem used in the questionnaire was “in my specific sport (e.g., basketball) classes”, and it was followed by 24 items grouped in six factors. These six factors, composed of four items, corresponded to autonomy satisfaction (e.g., “I feel a sense of choice and freedom in the things I undertake”); competence satisfaction (e.g., “I feel capable in what I do”); relatedness satisfaction (e.g., “I feel close and connected with other people who are important to me”); autonomy frustration (e.g., “most of the things I do feel like ‘I have to’”); competence frustration (e.g., “I have serious doubts that I can do the activities well”); and relatedness frustration (e.g., “I feel excluded from the group I want to belong to”). Responses were reported on a 5-point scale ranging from 1 (strongly disagree) to 5 (strongly agree). Cronbach’s alphas ranged from 0.71 to 0.78.

#### 2.2.2. Perceived Locus of Causality Scale (PLOC)

To measure students’ motivation in physical education classes, the Spanish version [50] of the perceived locus of causality scale (PLOC) [51] was used. The title that headed the questionnaire was “I participate in physical education classes”, and it was composed of 24 items. Specifically, there were four items per factor: intrinsic motivation (e.g., “because I enjoy learning new skills”), integrated regulation (e.g., “because I consider physical education is a part of me”), identified regulation (e.g., “because I want to get better at sports”), introjected regulation (e.g., “because I would feel bad about myself if I didn’t”), external regulation (e.g., “because those are the rules”), and demotivation (e.g., “but I really feel like I’m wasting my time”). The instrument used a Likert scale from 1 (strongly disagree) to 7 (strongly agree). Cronbach’s alphas ranged from 0.80 to 0.86.

#### 2.2.3. Behavioural Engagement

Students’ behavioural engagement was measured in the Spanish version [40] of the scale adapted from Shen, McCaughtry, Martin, Fahlman, and Garn [52]. The stem used in the questionnaire was “in PE classes”, and it was followed by 5 items addressing students’ perceptions of their effort, attention, and persistence in PE classes (e.g., “I work as hard as I can”). Responses were given on a 5-point scale ranging from 1 (strongly disagree) to 5 (strongly agree). Cronbach’s alpha was 0.87.

#### 2.2.4. Agentic Engagement

Agentic engagement was measured in the Spanish version [53] of the scale developed by Reeve [28]. This instrument is composed of five items that measure the construct of agentic engagement as a single factor (e.g., “During class, I share my preferences and opinions”). Responses were given on a 5-point scale ranging from 1 (strongly disagree) to 5 (strongly agree). Cronbach’s alpha was 0.72.

#### 2.2.5. Theoretical Knowledge

The test consisted of six multiple-choice questions in which the students were asked about the rules (e.g., “If a set is tied at 29 points, what happens?”), materials (e.g., “The shuttle used in official competitions is made of?”), and the basic game system. Additionally, they had a question in which they were asked to relate the types of strokes with their definition. Finally, they had to answer three open-ended questions about technical-tactical aspects: the progression of exercises (e.g., “Develop an exercise in which the serve and the net drop are worked”) and the anticipation of situations to gain advantage over the opponent (e.g., “What is the danger of a cross drop if the opponent is well positioned on the court?”).

#### 2.2.6. Practical Competence

To evaluate the different aspects related to racquet skills and technical strokes, a rubric was created. In the racquet skill part, the students were given four attempts to pick up the shuttle from the ground with the racquet, and in the second exercise, they had to keep the shuttlecock in the air for at least 10 s, giving hints. In the technical exercises part, they had to serve five times as if they were playing a singles match (they achieved the highest score if they made three good serves), they had to perform at least six consecutive clear strokes with a partner, and they had to perform at least six consecutive net-drop strokes with that partner. In the beginning of the intervention, as a way to evaluate the overall level of each condition, students were classified into three levels to carry out their learning activities. Their skill and ability with the racquet, both individually and in pairs, was the factor considered for this classification: low level, intermediate level, and high level (Supplementary Materials, Table S1). Figure S1 in the Supplementary Materials shows a graphical representation of the percentage of students corresponding to each level group.

### 2.3. Design and Procedure

The study design was a quasiexperimental pretest/posttest that aimed to compare the CBL experience (experimental group) and a more TT experience (control group). The main differences between these methodologies are shown in Table A1. Both groups (control and experimental) had the same PE teacher. The selection of participants used convenience sampling. To ensure a blinded process with the participants, the researchers shared with them that there was a research project to understand their perceptions of what occurred in PE classes, without telling that them there would be two conditions. The information and purpose of the project and the comparison between groups with different conditions were provided to the PE teacher. The inclusion criteria were two: class groups whose teacher accepted participating in the study and all the students who had attended more than 80% of the classes during the intervention.

The questionnaires were administered by a member of the research group before and after the intervention. The practical knowledge test was administered only after the intervention. The person who administered it also explained the purpose of the project, emphasized that the anonymity of the participants would be maintained, and encouraged the participants to give their most honest answers to the questions. The students completed the questionnaire in the classroom via a Google form in a climate that allowed them to concentrate without any distractions; its duration was about 20 min.

The study obtained approval from the Ethics Committee of a Spanish university. All participants were treated in agreement with the ethical guidelines of the American Psychological Association [54] with respect to consent, confidentiality, and the anonymity of their answers.

#### Description of the Intervention

The Template for Intervention Description and Replication (TIDIeR) checklist was used to describe the intervention [55]. The intervention aimed to teach badminton skills through a CBL experience. The purposes of this experience were to foster students’ learning and to improve students’ motivational outcomes. Although the students were taught differently, the sessions were delivered by the same teacher in both conditions. The intervention was carried out over a total of 10 sessions of 50 min, from April to June 2022, sessions that were part of their 2 weekly teaching hours. All classes were held at the sports facilities of the high school, specifically in the covered pavilion that had seven badminton courts.

**Figure A1:**
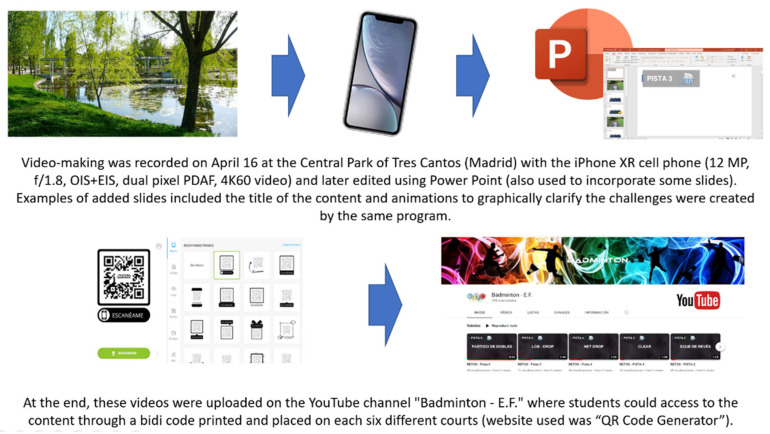
Creation process of the material used in the CBL sessions in the experimental group.

As shown in Table 1, within the DU created for the experimental group, 4 of the 10 sessions were designed under the CBL methodology. These 4 sessions were sessions 2, 3, 7, and 8. Figure A1 displays the creation process of the material used in the sessions.

The didactic objectives set for both groups were the same. However, the session objectives were modified for the experimental group in those sessions in which challenges were included.

### 2.4. Data Analysis

First, descriptive statistics (mean and standard deviation) and correlations among all the study variables were calculated. A Kolmogorov–Smirnov test was then performed to verify the normality of the data and show that the data were non-normally distributed (*p* < 0.05). Thus, nonparametric tests were used to analyse the differences between the groups. In order to test whether the groups behaved similarly before the intervention, Mann–Whitney U tests were performed to analyse the possible differences between them in terms of basic needs satisfaction and frustration, motivational regulations, and behavioural and agentic engagement. The initial level of practical competence was evaluated following the procedure described in Section 2.2.6 (Practical Competence). A Pearson chi-squared test was completed with the observation of standardized adjusted residuals and was used to assess the differences between the control and experimental groups in the distribution of students categorized as low, medium, and high level. Next, the main analysis was performed to investigate the intervention effects in two ways. First, to verify the intragroup differences between the pretest data collection and the posttest data collection, a Wilcoxon test was performed with each of the groups. Afterward, a new Mann–Whitney U test was conducted to analyse the intergroup differences between the two groups after the intervention. At this time, students’ scores both on knowledge acquisition and on practical competence in badminton after the intervention were included. The effect sizes of the comparisons were estimated by using Cliff’s delta. According to Vargha and Delaney [56], values between 0.11 and 0.28 should be considered as small; values between 0.28 and 43 should be considered as medium; and values higher than 0.43 should be considered as large. The SPSS 24.0 software program was used to process the data.

## 3. Results

### 3.1. Descriptive Statistics and Differences between Groups before the Intervention

The descriptive statistics and bivariate correlations are reported in Table 2. In general, the scores were high for basic psychological needs satisfaction, intrinsic motivation, integrated, and identified regulations; the scores were low for competence and relatedness frustration and for amotivation. Overall, the Pearson correlations showed significant and strong relationships between most of the study variables. Before the intervention, the satisfaction of basic psychological needs was related to the motivational variables in the expected direction, except for autonomy satisfaction and relatedness frustration before the intervention, in which no correlations were found. Positive and significant relations were also found between needs satisfaction and behavioural and agentic engagement. After the intervention, needs satisfaction was negatively related to needs frustration. Specifically, no correlations were found between autonomy satisfaction on one hand and competence and relatedness frustration on the other. In addition, autonomy satisfaction was positively related to introjected regulation, and no correlations were found between external regulation and amotivation. Each needs satisfaction was positively related to behavioural and agentic engagement. Finally, external regulation was negatively related to both types of engagement, while amotivation was only negatively related to agentic engagement.

As shown in Table 3, before the intervention, no significant differences were found between the control and experimental groups. As for practical competence, evaluated as described in Section 2.2.6, nonsignificant differences emerged between conditions according to participants’ levels (x^2^_2_ = 0.277, *p*. = 0.870).

### 3.2. Effects of the Intervention and Differences between Groups

Table 4 displays the effects of the intervention. For basic psychological needs, significant differences were found in each area of needs satisfaction, with a medium effect size according to Cliff’s delta. Related to students’ engagement, a higher score of behavioural engagement was found, also with a medium effect size.

The differences between the control and experimental groups are shown in Table 5. For basic psychological needs, the results showed that the experimental group had higher levels of autonomy and competence satisfaction. On the other hand, while needs frustration showed higher scores in the control group, there were no significant differences. In terms of intrinsic motivation, integrated and identified regulation showed higher levels in the experimental group after the intervention. Although the levels of introjected and external regulation and those of amotivation in the experimental group were higher both before and after the intervention in almost all the variables, the scores were lower after the intervention in this group. For students’ engagement, higher levels of both behavioural engagement and agentic engagement were found in the experimental group.

## 4. Discussion

The first aim of this study was to analyse how a CBL-based experience might affect students’ BPN satisfaction and frustration, motivation, behavioural engagement, and agentic engagement in comparison with the implementation of TT, all within a PE context. According to the study’s approach, the proposed hypotheses were partially fulfilled. On one hand, as hypothesized, students in the experimental group showed scores in their BPN satisfaction after the intervention. On the other hand, no significant differences were found in motivational regulations in the scores of the experimental group between before the CBL-based experience and after it. Participants in the experimental group showed higher levels of behavioural engagement after the intervention. However, no differences were found in terms of agentic engagement.

### 4.1. The Impact of CBL on Students’ BPN Satisfaction

Regarding BPN satisfaction, which had a medium effect size in terms of effects of the intervention, the present study findings align with the results from previous interventions carried out within the PE context, which have been successful in improving students’ BPN satisfaction [4,34,37,41]. According to the literature, students’ BPN satisfaction can be fostered through the implementation of interventions specifically designed to create motivational contexts [31,32,41] or through model-based experiences [34,35,38,39]. In the case of the former group of studies, the improvement of motivational outcomes (e.g., BPN satisfaction) follows a more straightforward mechanism: an intervention that is designed on the basis of specific strategies that have been found to be valid to make students feel more autonomous, competent, and satisfied with their relationships has proven to be actually effective. In the case of the latter group of studies, several reasons have been identified that might explain why certain methodological approaches can improve students’ NPB satisfaction. Aspects such as students’ feelings about being on a team, the management of possible conflicts, the opportunity for planning and scheduling activities, the provision of feedback, and the use of coevaluation or shared evaluation, which are essential features in the sport education model, could be significantly affecting students’ feelings of autonomy, competence, and relatedness satisfaction [37].

Along the same line, elements such as the opportunity to make decisions independently of the teacher, questioning and debating ideas, and the role of the teacher as a guide or facilitator, which are representative of the TGFU methodology, are likely to foster students’ autonomy and competence satisfaction [35].

The present study is the first attempt to test whether a CBL experience in PE might have an effect on students’ motivational outcomes. According to the present study findings, characteristics of this pedagogical approach, such as suggesting individual progression, offering choice, or identifying students’ interests, might be relevant for the students to feel that they are autonomous in their PE classes. Additionally, the fact that CBL promotes the provision of optimal challenges or the clear establishment of standards and evaluation criteria [39,41,47] can explain why students who are taught according to this methodology are more likely to feel competent than their peers taught by a more TT methodology. Lastly, aspects such as the development of cooperative activities where the students collaborate in pursuit of the established objectives could be involved in the feeling of relatedness [57]. Overall, these findings point out that CBL might be another promising methodological approach to positively affect students’ motivational patterns.

### 4.2. The Impact of CBL on Students’ Motivational Regulations

Surprisingly, no significant differences were found in any of the motivational regulations after the intervention. According to the SDT tenets, BPN is considered a more proximal construct than motivational regulations in the explanation of human behaviour [1]. In other words, the satisfaction or frustration of BPN will lead to certain motivational regulations. Perhaps, while affecting students’ BPN satisfaction as the most proximal psychological construct, the cross-sectional nature of the present study did not allow for detecting the potential and expected influence of this experience on students’ motivational regulations. Along this line, the studies exploring the effects of interventions on motivational patterns have suggested that needs satisfaction might be more malleable than motivational regulations (e.g., [41,52]) in that the teaching strategies used in the PE class are specifically designed to alter students’ BPN [15]. On the other hand, while there is no previous evidence establishing methodological considerations regarding the duration of school-based interventions [58], other studies have also highlighted the importance that the length of interventions may have on motivational outcomes [34,59,60] given that shorter interventions might reflect effects only in the more proximal construct of the model (BPN satisfaction) and given that longer interventions might also reveal effects in the more distal part of the sequence (motivational regulations). Further research could explore the role played by time in the malleability of different motivational outcomes in education through the implementation of longitudinal designs.

### 4.3. The Impact of CBL on Students’ Engagement

The findings of the present study revealed that students’ behavioural engagement improved after the intervention, showing a medium effect size. The present study findings align with previous works that have pointed out a positive association between the way teachers interact with their students and how engaged they are in the classroom (e.g., [7,21]). Specifically, it has been suggested that needs-supportive teaching behaviours have a positive influence on students’ behavioural engagement [24,25]. In the case of the present study, it is plausible to think that the support for competence satisfaction through the provision of optimal challenges and clear guidance might make students more likely to be active, put more effort into their tasks, participate, or follow the teachers’ instructions. In other words, certain CBL features could be effective tools in the improvement of students’ behavioural engagement. Given the positive association between students’ behavioural engagement and other performance and learning outcomes [17,22], it is safe to highlight the potential of CBL to improve educational processes.

On the other hand, agentic engagement did not significantly differ in the experimental group between before the intervention and after it. There have been several attempts to explore agentic engagement from an SDT perspective [26,27]. This line of research has revealed that autonomy support can positively influence agentic engagement. When teachers appreciate, encourage, and enthusiastically invite students’ input and initiative, students might respond in kind and become more likely to speak up and offer their input [27]. While, according to the findings, the CBL intervention implemented in the present work can be seen as a needs-supportive experience for students, the experience does not seem to foster students’ agentic engagement. Reeve and Shin [26] point out that students can show agentic engagement through certain actions, such as expressing preferences, making suggestions, asking for a say in what they do, or asking for needed resources. However, there are some features of the CBL approach that, relevant as they might be to improve students’ competence, could be playing a detrimental role in nourishing the experience of agentic engagement. The fact that students are told to engage in close challenges entirely designed by the teacher might have made it difficult for the students to have a say in what they do, or the provision of abundant graphical support from the beginning might have precluded students in the experimental group from asking the teacher for extra help and resources. While the features of this CBL experience better resemble the autonomy-supportive style rather than the control-motivating style described by Reeve and Shin [26], it would be interesting to explore a way of adapting certain CBL features so that this approach could foster students’ agentic engagement.

### 4.4. The Impact of CBL on Students’ Performance

Finally, another interesting finding was the slightly positive impact that the intervention had on badminton-specific motor skills. At the end of the intervention, the teacher assessed students’ badminton-specific motor skills in both groups through different activities to check the acquisition of skills. Although these results were not significant, the increase by almost one point in students in the experimental condition could be related to the significant improvement of competence satisfaction among these students. If students believe that the activities are more challenging and that they can establish their own goals, receive feedback and praise about their improvement and effort, or receive clear expectations and hints to foster a deeper understating, they are more likely to engage in the activities and, in turn, improve their level of practical competence. These findings aligned with those indicating that perceived competence might be related to the learning process [42,44,45]. These studies suggested that the provision of choice and the promotion of active participation between students in CBL experiences might significantly improve learning and enhance their competence satisfaction, which might also be related to students’ engagement.

### 4.5. Limitations and Future Lines of Research

This study had some limitations that are worth noting. First, practical competence and theoretical knowledge were assessed only after the intervention. Although some information was collected before the intervention, which suggests a similar initial level in terms of competence level, future studies should use the same measure to assess these variables both before the intervention and after it. Second, another limitation concerns the sample size of both the control and experimental groups, so the statistical significance was hard to obtain. The small group sizes were due to the difficulties inherent in conducting an intervention study within a PE context. It would be interesting to use a large sample size for future research to allow us to conduct a predictive statistical analysis to explore the interplay between the different variables in both groups. Third, the PE teacher that took part in the study was provided with the purpose of the study and information about the comparison between the groups under different conditions. Finally, the length of the intervention could be a limitation on the effects on motivational regulations. Future studies could explore the effects of longer CBL experiences on both students’ and teachers’ motivational outcomes owing to the demonstrated relationship between positive learning outcomes and autonomy-supportive teaching behaviours. A recent study has pointed out the association between this teaching style and novelty satisfaction, which, in turn, predicted students’ physical activity intention [57]. This association leads us to think that the CBL methodology, as an innovative approach, could enhance not only competence but also novelty, resulting in improved levels of significant learning and physical activity practice. Further research on analysing the effects of CBL experiences on BPN and novelty satisfaction would be also interesting.

## 5. Conclusions

This study aimed to examine the influence of a CBL intervention on students’ motivation, engagement, and performance in PE. The findings of the study highlight the CBL model’s potential to foster students’ BPN satisfaction and suggest that students might improve their sport-specific motor skills when implementing this methodology. Similarly, it seems that the CBL approach can promote students’ behavioural engagement. Overall, the present work suggests that embracing the key features of CBL can be a promising avenue for improving PE settings from a motivational perspective.

## Figures and Tables

**Table 1:** Summary of the sessions’ contents for control condition and experimental condition.

	Traditional Teaching Condition	Challenge-Based Learning Condition
Session 1	Both groups engaged in the same activities, with the target of familiarizing themselves with the materials and the most basic elements of the sport.	
Session 2	They began to work on the most basic technical skills (such as forehand and backhand low-handed strokes) by following the teacher’s instructions.	They began to work on the most basic technical skills (such as forehand and backhand low-handed strokes) by using different challenge cards.
Session 3	Students learned to serve by working individually and repeating the technical gesture over and over.	They learned to serve by working in pairs, using cards that progressed from level one to level four that they had to complete.
Sessions 4, 5 and 6	They worked on the different badminton strokes (net drop, lob, clear, drop, and smash) while using the method, teaching strategies and techniques, and groupings described for the traditional methodology.	They worked on the different badminton strokes (net drop, lob, clear, drop, and smash) while using the method, teaching strategies and techniques, and groupings described for the CBL methodology.
Sessions 7 and 8	They reviewed all the elements seen. They continued with the same dynamics as those from the previous sessions.	They reviewed all the elements seen. A challenge activity was designed to work autonomously thanks to the inclusion of QR codes that linked each track to different technical-tactical videos.
Session 9	Singles competition.	Mixed doubles competition.
	Practical test (practical competence)	
Session 10	Theoretical test (theoretical knowledge)	

**Table 2:** Descriptive statistics and bivariate correlations of the study variables.

	1	2	3	4	5	6	7	8	9	10	11	12	13	14	15	16	17	18	19	20	21	22	23	24	25	26	27	28
1. PRE autonomy satisfaction	1	0.29 *	0.40 **	−0.58 **	−0.28 *	−0.15	0.50 **	0.40 **	0.57 **	0.30 *	−0.09	−0.25	0.49 **	0.67 **	0.74 **	0.39 **	0.41 **	−0.35 *	−0.30 *	−0.35 *	0.34 *	0.32 *	0.44 **	0.31 *	−0.22	−0.10	0.51 **	0.56 **
2. PRE competence satisfaction		1	0.55 **	−0.61 **	−0.75 **	−0.44 **	0.65 **	0.65 **	0.58 **	0.20	−0.04	−0.40 **	0.50 **	0.44 **	0.26	0.86 **	0.55 **	−0.41 **	−0.55 **	−0.40 **	0.55 **	0.54 **	0.61 **	0.17	−0.42 **	−0.38 **	0.59 **	0.53 **
3. PRE relatedness satisfaction			1	−0.51 **	−0.63 **	−0.79 **	0.56 **	0.33 *	0.48 **	0.27	−0.00	−0.58 **	0.35 *	0.52 **	0.30 *	0.46 **	0.76 **	−0.29 *	−0.32 *	−0.65 **	0.49 **	0.27	0.50 **	0.35 *	−0.30 *	−0.41 **	0.42 **	0.59 **
4. PRE autonomy frustration				1	0.49 **	0.28 *	−0.76 **	−0.62 **	−0.65 **	−0.43 **	0.18	0.46 **	−0.45 **	−0.55 **	−0.56 **	−0.60 **	−0.49 **	0.61 **	0.42 **	0.42 **	−0.64 **	−0.52 **	−0.57 **	−0.25	0.54 **	0.41 **	−0.56 **	−0.73 **
5. PRE competence frustration					1	0.63 **	−0.44 **	−0.46 **	−0.35 *	00.08	00.18	0.50 **	−0.46 **	−0.39 **	−00.27	−0.69 **	−0.68 **	0.33 *	0.76 **	0.71 **	−0.48 **	−0.38 **	−0.41 **	00.07	0.50 **	0.55 **	−0.42 **	−0.57 **
6. PRE relatedness frustration						1	−0.21	−0.03	−0.13	0.03	−0.00	0.45 **	−0.18	−0.24	−0.02	−0.27	−0.70 **	0.16	0.37 **	0.66 **	−0.23	0.01	−0.20	0.01	0.29 *	0.43 **	−0.15	−0.39 **
7. PRE intrinsic motivation							1	0.82 **	0.91 **	0.59 **	−0.05	−0.43 **	0.35 *	0.66 **	0.53 **	0.62 **	0.54 **	−0.43 **	−0.37 **	−0.40 **	0.81 **	0.72 **	0.78 **	0.55 **	−0.36 *	−0.28	0.62 **	0.67 **
8. PRE integrated regulation								1	0.83 **	0.39 **	−0.09	−0.29 *	0.36 *	0.60 **	0.49 **	0.67 **	0.40 **	−0.36 *	−0.32 *	−0.23	0.69 **	0.79 **	0.76 **	0.40 **	−0.37 **	−0.18	0.60 **	0.58 **
9. PRE identified regulation									1	0.55 **	−0.04	−0.31 *	0.37 **	0.72 **	0.57 **	0.56 **	0.55 **	−0.38 **	−0.18	−0.28	0.69 **	0.70 **	0.83 **	0.55 **	−0.30 *	−0.16	0.59 **	0.57 **
10. PRE introjected regulation										1	0.45 **	−0.01	0.12	0.37 **	0.38 **	0.18	0.09	−0.17	0.08	−0.00	0.31 *	0.33 *	0.39 **	0.76 **	0.20	0.12	0.36 **	0.28 *
11. PRE external regulation											1	0.38 **	−0.34 *	−0.03	0.03	−0.07	−0.19	0.21	0.17	0.07	−0.05	0.01	−0.18	0.27	0.46 **	0.14	−0.24	−0.09
12. PRE amotivation												1	−0.37 **	−0.21	−0.04	−0.33 *	−0.40 **	0.33 *	0.38 **	0.44 **	−0.39 **	−0.24	−0.38 **	0.02	0.36 *	0.59 **	−0.37 **	−0.33 *
13. PRE behavioural engagement													1	0.42	0.30	0.45	0.31	−0.27	−0.35	−0.21	0.28	0.24	0.45	0.18	−0.26	−0.18	0.79	0.41
14. PRE agentic engagement														1	0.66 **	0.46 **	0.56 **	−0.35 *	−0.33 *	−0.40 **	0.49 **	0.46 **	0.56 **	0.40 **	−00.26	−00.12	0.51 **	0.75 **
15. POST autonomy satisfaction															1	0.44 **	0.39 **	−0.40 **	−0.25	−0.26	0.50 **	0.45 **	0.50 **	0.34 *	−0.22	−0.09	0.46 **	0.65 **
16. POST competence satisfaction																1	0.50 **	−0.42 **	−0.58 **	−0.32 *	0.60 **	0.62 **	0.56 **	0.16	−0.41 **	−0.28 *	0.54 **	0.57 **
17. POST relatedness satisfaction																	1	−0.32 *	−0.36 **	−0.75 **	0.55 **	0.39 **	0.57 **	0.14	−0.37 **	−0.36 *	0.43 **	0.60 **
18. POST autonomy frustration																		1	0.34 *	0.22	−0.36 **	−0.38 **	−0.39 **	−0.13	0.70 **	0.48 **	−0.46 **	−0.45 **
19. POST competence frustration																			1	0.49 **	−0.35 *	−0.38 **	−0.15	0.18	0.47 **	0.45 **	−0.29 *	−0.45 **
20. POST relatedness frustration																				1	−0.44 **	−0.22	−0.29 *	00.06	0.41 **	0.51 **	−0.26	−0.49 **
21. POST intrinsic motivation																					1	0.70 **	0.75 **	0.37 **	−0.31 *	−0.35 *	0.56 **	0.64 **
22. POST integrated regulation																						1	0.69 **	0.35 *	−0.31 *	−0.34 *	0.50 **	0.47 **
23. POST identified regulation																							1	0.54 **	−0.31 *	−0.26	0.69 **	0.57 **
24. POST introjected regulation																								1	0.23	0.24	0.44 **	0.32 *
25. POST external regulation																									1	0.58 **	−0.32 *	−0.40 **
26. POST amotivation																										1	−0.22	−0.30 *
27. POST behavioural engagement																											1	0.55 **
28. POST agentic engagement																												1
M (SD)	3.07	3.94	3.93	3.06	2.29	1.88	4.67	4.27	4.74	3.88	4.05	2.65	4.04	4.15	3.22	4.00	4.11	3.17	2.30	1.89	4.96	4.59	4.98	3.99	4.31	2.88	4.20	4.49
	(0.80)	(0.82)	(0.88)	(0.98)	(1.00)	(0.89)	(1.37)	(1.61)	(1.39)	(1.42)	(1.17)	(1.12)	(0.64)	(1.38)	(0.71)	(0.81)	(0.77)	(0.96)	(1.07)	(0.76)	(1.31)	(1.40)	(1.45)	(1.31)	(1.23)	(1.30)	(0.61)	(1.49)

Note: * *p* < 0.05; ** *p* < 0.01.

**Table 3:** Differences between groups before the intervention.

	Control Group (*n* = 24) M (SD)	Experimental Group (*n* = 26) M (SD)	Z	*p*	Cliff’s Delta
Autonomy satisfaction	2.98 (0.73)	3.15 (0.86)	−0.605	0.545	0.08
Competence satisfaction	3.86 (0.64)	4.01 (0.97)	−1.050	0.294	0.13
Relatedness satisfaction	4.00 (0.72)	3.86 (1.02)	−0.225	0.822	0.03
Autonomy frustration	3.21 (0.98)	2.92 (0.99)	−1.160	0.246	0.15
Competence frustration	2.23 (0.79)	2.34 (1.17)	−0.107	0.915	0.01
Relatedness frustration	1.70 (0.70)	2.04 (1.01)	−1.129	0.259	0.15
Intrinsic motivation	4.47 (1.26)	4.85 (1.47)	−1.120	0.263	0.15
Integrated regulation	4.04 (1.51)	4.48 (1.69)	−0.983	0.326	0.13
Identified regulation	4.64 (1.22)	4.84 (1.54)	−0.487	0.626	0.06
Introjected regulation	3.41 (1.24)	4.32 (1.45)	−2.454	0.014	0.33
External regulation	3.86 (1.18)	4.21 (1.16)	−0.741	0.459	0.06
Amotivation	2.49 (1.22)	2.79 (1.01)	−1.328	0.184	0.19
Behavioural engagement	3.96 (0.60)	4.12 (0.67)	−0.878	0.380	0.12
Agentic engagement	4.05 (1.23)	4.25 (1.53)	−0.302	0.763	0.04

**Table 4:** Comparison of the effects of the intervention.

		Control Group (*n* = 24)			Experimental Group (*n* = 26)		
		M (SD)	Z	Cliff’s Delta	M (SD)	Z	Cliff’s Delta
Autonomy satisfaction	Pre	2.98 (0.73)	−0.549	0.07	3.15 (0.86)	−1.962	0.26 *
	Post	3.03 (0.73)			3.39 (0.66)		
Competence satisfaction	Pre	3.86 (0.64)	−0.980	0.13	4.01 (0.97)	−2.440	0.33 *
	Post	3.79 (0.68)			4.18 (0.89)		
Relatedness satisfaction	Pre	4.00 (0.72)	−1.298	0.17	3.86 (1.02)	−2.306	0.32 *
	Post	4.17 (0.62)			4.06 (0.89)		
Autonomy frustration	Pre	3.21 (0.98)	−0.565	0.07	2.92 (0.99)	−0.046	0.01
	Post	3.43 (0.90)			2.92 (0.97)		
Competence frustration	Pre	2.23 (0.79)	−1.018	0.13	2.34 (1.17)	−0.433	0.06
	Post	2.34 (0.90)			2.25 (1.22)		
Relatedness frustration	Pre	1.70 (0.70)	−0.096	0.01	2.04 (1.01)	−0.041	0.01
	Post	1.80 (0.70)			1.97 (0.82)		
Intrinsic motivation	Pre	4.47 (1.26)	−1.670	0.23	4.85 (1.47)	−1.256	0.18
	Post	4.79 (1.32)			5.12 (1.31)		
Integrated regulation	Pre	4.04 (1.51)	−1.741	0.24	4.48 (1.69)	−1.503	0.21
	Post	4.42 (1.46)			4.74 (1.34)		
Identified regulation	Pre	4.64 (1.22)	−1.417	0.21	4.84 (1.54)	−1.513	0.21
	Post	4.83 (1.40)			5.12 (1.51)		
Introjected regulation	Pre	3.41 (1.24)	−1.767	0.24	4.32 (1.45)	−0.556	0.07
	Post	3.80 (1.27)			4.15 (1.34)		
External regulation	Pre	3.86 (1.18)	−2.444	0.33 *	4.21 (1.16)	−0.212	0.03
	Post	4.43 (1.36)			4.19 (1.11)		
Amotivation	Pre	2.49 (1.22)	−1.313	0.18	2.79 (1.01)	−0.449	0.06
	Post	2.85 (1.35)			2.89 (1.29)		
Behavioural engagement	Pre	3.96 (0.60)	−0.846	0.12	4.12 (0.67)	−2.573	0.35 *
	Post	4.03 (0.60)			4.36 (0.59)		
Agentic engagement	Pre	4.05 (1.23)	−1.801	0.25	4.25 (1.53)	−1.869	0.25

Note: * *p* < 0.05.

**Table 5:** Differences between groups after the intervention.

	Control Group (*n* = 25) M (SD)	Experimental Group (*n* = 25) M (SD)	Z	*p*	Cliff’s Delta
Autonomy satisfaction	3.03 (0.73)	3.39 (0.66)	−1.607	0.108	0.22
Competence satisfaction	3.79 (0.68)	4.18 (0.89)	−2.203	0.028	0.30
Relatedness satisfaction	4.17 (0.62)	4.06 (0.89)	−0.167	0.868	0.02
Autonomy frustration	3.43 (0.90)	2.92 (0.97)	−1.912	0.056	0.26
Competence frustration	2.34 (0.90)	2.25 (1.22)	−0.780	0.435	0.11
Relatedness frustration	1.80 (0.70)	1.97 (0.82)	−0.607	0.544	0.08
Intrinsic motivation	4.79 (1.32)	5.12 (1.31)	−0.926	0.354	0.12
Integrated regulation	4.42 (1.46)	4.74 (1.34)	−0.613	0.540	0.08
Identified regulation	4.83 (1.40)	5.12 (1.51)	−0.751	0.453	0.10
Introjected regulation	3.80 (1.27)	4.15 (1.34)	−1.012	0.311	0.13
External regulation	4.43 (1.36)	4.19 (1.11)	−1.277	0.202	0.18
Amotivation	2.85 (1.35)	2.89 (1.29)	−0.127	0.899	0.01
Behavioural engagement	4.03 (0.60)	4.36 (0.59)	−2.042	0.041	0.27
Agentic engagement	4.38 (1.41)	4.59 (1.58)	−0.516	0.606	0.07
Theoretical knowledge	6.48 (2.21)	6.79 (2.35)	−0.515	0.606	0.07
Practical competence	6.85 (1.53)	7.65 (2.24)	−1.68	0.093	0.22

## Data Availability

Not applicable.

## Supplementary Materials

The following supporting information can be downloaded at https://www.mdpi.com/article/10.3390/ejihpe13040052/s1, Figure S1: Percentage of students corresponding to each level group; Table S1: Entry level indicators.

## Appendix A

**Table A1:** Features of the two methodological approaches used in the control group and the experimental group.

	Traditional Teaching Condition	Challenge-Based Learning Condition
Didactic Unit structure	Familiarization with the materials and spaces of the sport/Learning the most basic technical-tactical elements of the sport/review/competition/practical exam and the theoretical exam	
Lesson structure	Pick up and welcome/warming/main part/return to peace/closing and farewell	
Methods	The focus is on the result, seeking to minimize errors	The focus is on the process, seeking trial and error
Teaching strategies	Pure analytics/progressive analytics	Pure global/global polarizing attention
Teaching techniques	It is based on the existence of a concrete solution to a motor problem that the teacher establishes as a model to be followed (reproduction of models)	The student participates intellectually, searching for solutions to the posed problems
Teaching styles	Modified direct command and task assignment	Guided discovery, problem solving, level groups, reciprocal teaching, and socializing style
Grouping	Individual activities or activities in pairs	Individual activities, activities in pairs, activities in threesomes, or activities in large groups, for the promotion of coeducation
Individualized learning	The activities are identical for all the students, so they have to follow the same pace	The students are free to move forward from challenge to challenge according to their level and skills without needing to complete them all
Tasks presentation	The teacher presents the activities just before practising them, by doing the exercises themself	Challenges are presented with the support of graphical resources (images, videos); all the challenges are available from the beginning
Students’ autonomy support	Students cannot choose the activities they are involved in	Students can choose the challenges that they want to tackle according to their own perceived competence
Teacher’s role	The teacher is in charge of explaining each new activity and giving feedback to the students	Because the challenges are presented to students through visual resources (cards, pictures, videos, etc.), the teacher is free to better support and give feedback to the students during the practice
Students’ involvement in their evaluation	Students are not involved in their evaluation	Students take part in part of their evaluation because they can monitor their performance by achieving different challenges, and self-assessment sheets are provided
Collaborative work	There is no presence of collaborative work	Some of the activities or challenges can be achieved only by collaborating with other students
Use of TIC	No use of ICTs	Use of smartphones for video viewing and challenges
