# Supplementary material for: The Impact of a Challenge-Based Learning Experience in Physical Education on Students’ Motivation and Engagement
Source: Eur J Investig Health Psychol Educ. 2023 Mar 26;13(4):684–700. doi: 10.3390/ejihpe13040052 (PMC10137837; doi:10.3390/ejihpe13040052)

Table S1. Entry level indicators.

Low Level	Intermediate Level	High Level
The student has difficulty keeping the steering wheel in play (in the air).<br>The student is not able to change grip.<br>The student shows difficulty in lifting the shuttlecock off the ground.	The student is able to keep the shuttlecock in the game even if it occasionally falls to the ground.<br>The student is able to change grip in some cases.<br>The student is able to lift the shuttlecock off the ground with the racquet even if he/she does not maintain control of the shuttlecock when doing so and eventually drops it to the ground on occasion.	The student is able to keep the shuttlecock in the air both when playing individually and when playing with a partner.<br>The student is able to change grip depending on whether he/she has to hit forehand or backhand.<br>The student is able to lift the shuttle off the ground with the racquet.

Figure S1. Percentage of students corresponding to each level group.

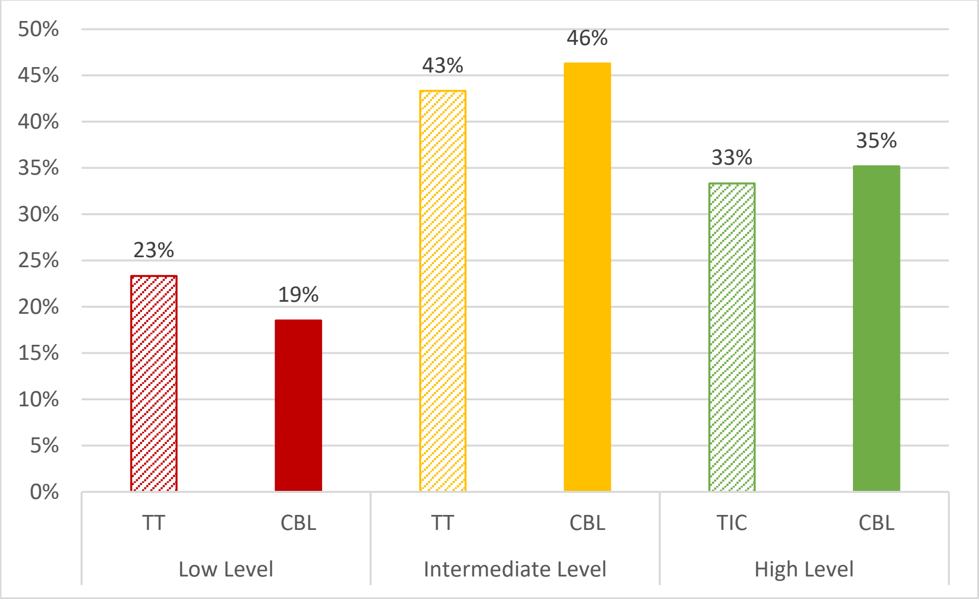

Supplement: Supplementary file 1 [file ejihpe-13-00052-s001.zip › ejihpe-2180441-supplementary.pdf]
